# Therapeutic Targeting Notch2 Protects Bone Micro-Vasculatures from Methotrexate Chemotherapy-Induced Adverse Effects in Rats

**DOI:** 10.3390/cells11152382

**Published:** 2022-08-02

**Authors:** Yaser Peymanfar, Yu-Wen Su, Mohammadhossein Hassanshahi, Cory J. Xian

**Affiliations:** UniSA Clinical and Health Sciences, University of South Australia, Adelaide, SA 5001, Australia; yaser.peymanfar@mymail.unisa.edu.au (Y.P.); yu-wen.su@unisa.edu.au (Y.-W.S.); mohammadhossein.hassanshahi@sahmri.com (M.H.)

**Keywords:** methotrexate, cancer chemotherapy, bone vasculature, notch signalling

## Abstract

Intensive cancer chemotherapy is well known to cause bone vasculature disfunction and damage, but the mechanism is poorly understood and there is a lack of treatment. Using a rat model of methotrexate (MTX) chemotherapy (five once-daily dosses at 0.75 mg/kg), this study investigated the roles of the Notch2 signalling pathway in MTX chemotherapy-induced bone micro-vasculature impairment. Gene expression, histological and micro-computed tomography (micro-CT) analyses revealed that MTX-induced micro-vasculature dilation and regression is associated with the induction of Notch2 activity in endothelial cells and increased production of inflammatory cytokine tumour necrosis factor alpha (TNFα) from osteoblasts (bone forming cells) and bone marrow cells. Blockade of Notch2 by a neutralising antibody ameliorated MTX adverse effects on bone micro-vasculature, both directly by supressing Notch2 signalling in endothelial cells and indirectly via reducing TNFα production. Furthermore, in vitro studies using rat bone marrow-derived endothelial cell revealed that MTX treatment induces Notch2/Hey1 pathway and negatively affects their ability in migration and tube formation, and Notch2 blockade can partially protect endothelial cell functions from MTX damage.

## 1. Introduction

Methotrexate (MTX) chemotherapy is commonly used in paediatric oncology for the treatments of childhood acute lymphoblastic leukemia (ALL) and osteosarcoma, and in adults it is used for the treatment of non-Hodgkin’s lymphoma, breast cancer and osteosarcoma [1,2]. MTX as an anti-metabolite agent acts by inhibiting the enzyme dihydrofolate reductase, therefore blocking the thymidylate and purine synthesis in cells [3]. Although chemotherapy has elevated the rates of success in cancer treatment, it has been accompanied by long-term adverse effects on the bone such as osteoporosis, osteonecrosis, increased risks of bone fractures and haematopoietic defects [4,5]. In addition, several studies have reported chemotherapy induced-bone marrow micro-vasculature injuries and haemorrhage among cancer patients and survivors [6,7,8].

Bone vasculature has crucial roles in bone turnover and bone homeostasis by supplying oxygen, micronutrients, growth factors and cytokines, and transferring precursor cells for osteoblasts (bone forming cells) and osteoclasts (bone resorbing cells) as well as waste products [9]. In long bones, blood flows via a network of vessels in cortical bone canals which then enter the medullary cavity to supply bone marrow and trabecular bone [10]. Recent findings have also illustrated a network of micro-vessels in the cortical bone connecting endosteal and periosteal circulations [11]. Many studies have also indicated important contributions of cortical bone vasculature canal porosity in bone strength during diseases and aging [12,13]. Furthermore, in the bone marrow, findings have revealed presence of diverse types of endothelial cells based on cell surface markers, including type H capillaries which are suggested to play active roles in bone formation (osteogenesis) and type L sinusoids which mostly interact with haematopoietic stem cells [14]. In the bone marrow, sinusoidal endothelial cells line the mono-layer sinusoids which are unique in structure and function and are more likely to be adversely affected following irradiation or chemotherapy [15,16]. Recent studies using rat models of MTX chemotherapy have shown bone marrow sinusoidal vasculature dilation and increased permeability and apoptosis of endothelial cells [17]. However, the underlying molecular mechanisms behind the MTX chemotherapy-induced bone micro-vasculature damage and recovery remain largely unclear. 

Notch signalling plays a pivotal role in controlling cell communication and regulation of the development and homeostasis of many organs/tissues including the cardiovascular system [18]. The binding of Notch ligands Jagged (Jag)1, Jag2, Delta-like (Dll)1, Dll3 and Dll4 to the receptors (Notch 1–4) of neighbouring cells activates series of enzymatic cleavage by disintegrin and metalloprotease ADAM family member (a TNFα-converting enzyme) and secondary cleavage by γ-secretase complex. As a result, the Notch intracellular domain (NICD) is released into the cytoplasm, which then translocates to the nucleus and leads to the expression of Notch target genes, including hairy enhancer of split (Hes) and Hes-related YRPW motif (Hey) [19,20]. Evidence has shown that Notch signalling is involved in the proliferation, differentiation, and cell fate decision in bone, neuronal, epithelial and endothelial cells [18]. It has been shown that endothelial cells express all four Notch receptors, with redundancy in Notch1 and Notch4 expression [18]. Notch pathway has been implicated in angiogenesis and vascular homeostasis [21] and it can modulate endothelial cell survival and functions through interacting with other angiogenic pathways such as vascular endothelial growth factor (VEGF) [22], nitric oxide (NO) [23,24], basic fibroblast growth factor (bFGF) [25] and hypoxia-inducible factor 1α [26]. Deregulation in Notch signalling has been linked to several inheritable vascular disorders such as Alagille syndrome (AGS), cerebral autosomal dominant arteriopathy with subcortical infarcts and leukoencephalopathy (CADASIL) [20] and vascular pathology [18]. In addition, cross talk between Notch pathway and inflammatory cytokines was shown to play an important role in endothelial functionality [27,28,29,30], and Notch genetically modified models and pharmacological Notch inhibitors have been reported to control vasculature dysfunction in atherosclerosis [31] and rheumatoid arthritis (RA) [32]. 

Despite the very few pieces of evidence about the roles of Notch2 in endothelial cell function [21,27], the roles of Notch2 in MTX chemotherapy-induced bone micro-vasculature damage is unknown. The current study used a rat model of MTX chemotherapy to investigate the roles of Notch2 signalling in MTX treatment-induced bone micro-vasculature damage. This study showed that administration of specific anti-Notch2 neutralising antibody may protect bone micro-vasculature from MTX-induced dilation and regression in rats and may preserve endothelial cell functionality following MTX treatment in vitro. 

## 2. Materials and Methods

### 2.1. Rat MTX Chemotherapy Time-Course Study

Six-week-old male Sprague Dawley rats (120–150 g approximately) were injected subcutaneously with MTX (Sigma-Aldrich, Castle Hill, NSW, Australia) at 0.75 mg/kg/day once daily for 5 days, mimicking the intensive treatment of acute lymphoblastic leukemia [33,34]. A group of saline-injected rats was used as a control group. Bone specimens were collected for histological and gene expression analyses on days 6, 9 and 14 following the first injection (n = 5 rats/group). The protocol and regulatory aspects for rodent experimentation procedures were approved by the institutional Animal Ethics Committee.

### 2.2. MTX and Anti-Notch2 Antibody Treatment Animal Trial

Six-week-old male Sprague Dawley rats (around 120–150 g) received placebo or MTX subcutaneously as described above. To examine the roles of Notch2 signalling in MTX treatment-induced changes in cortical vascular porosity and bone marrow vasculature, rats were randomly divided into four groups receiving: Saline+Control IgG; Saline+Anti-Notch2 antibody; MTX+Control IgG; and MTX+Anti-Notch2 antibody (n = 10 rats in each group). Anti-ragweed antibody as the control IgG and anti-Notch2 antibody (against the negative regulatory region of Notch2 or NRR2) (kindly supplied by Genentech, South San Francisco, CA, CA) were administrated through intraperitoneal injection (5 mg/kg) at days 1, 4 and 7 following the first MTX injection [35]. This dose of antibody was previously found as an effective dose for blocking Notch2 receptor without causing gastrointestinal toxicity [36,37,38]. The animal study was approved by the institutional Animal Ethics Committee.

Tibial bone specimens were collected at day 9 (after the first MTX injection), which is a time point shown with most significant histological damages in bone and bone marrow following MTX treatment [33,35]. From the n = 10 rats/group, samples from n = 6 rats were used for histological, immunohistochemical and gene expression analyses, and n = 4 for micro-computed tomography (micro-CT) analyses (Skyscan 1276, Bruker, Kontich, Belgium). Following euthanasia by CO2, the proximal left tibia were fixed in 10% formalin for 24 h, decalcified in Immunocal (Decal Corp, Tallman, NY, USA) for 21 days at 4 °C and processed and embedded in paraffin for histology and immunohistochemistry analyses (1). Proximal right tibia were collected and snap frozen in liquid nitrogen and stored at −80 °C for gene expression analyses [39]. For the micro-CT imaging, tibias were collected and stored in 80% ethanol until scanning with micro-CT (see below). 

### 2.3. Histological Analyses of Bone Marrow Sinusoids

To assess MTX damages on BM sinusoidal endothelium and potential role of anti-Notch2 antibody treatment, sections of 4 µm from paraffin-embedded tibial specimens were mounted on Superfrost^TM^ slides (Thermo Fisher Scientific Australia, Scoresby, VIC, Australia). Sections were stained by haematoxylin and eosin (H&E), viewed, and captured by Olympus BX41 light microscope (Olympus, Melbourne, VIC, Australia). Images were analysed by Image J software (National Institute of Health, Bethesda, MD), and bone marrow sinusoids were morphologically recognised by their typical irregular shapes with thin walls consisting of monolayered endothelial cells [16]. Sinus area/marrow area (µm^2^) and sinusoidal diameter (µm) were measured in the secondary spongiosa of tibia sections using the image analysis software as described [40]. 

### 2.4. Immunohistochemical Analyses

To assess treatment effects on protein expression of inflammatory cytokine tumour necrosis factor alpha (TNFα) in bone marrow collected from control and treatment group of rats, immunostaining was conducted using previously published protocol [41]. To localise protein expression of activated Notch2 (Notch2 intracellular domain, NICD2) in the bone and to examine treatment effects, Notch2 immunostaining was performed. Briefly, sections were deparaffinised, hydrated, and were quenched in 3% H_2_O_2_ and incubated in citrate buffer (pH6) for antigen retrieval process. Blocking was conducted with 10% rabbit serum in PBS for TNFα staining, or with 5% pig serum in 4% BSA and 0.1% Triton-X 100 plus 0.05% Tween 20 in PBS for Notch2 staining for 60 min at room temperature. Then, sections were incubated with goat polyclonal primary antibodies against TNFα (Santa Cruz Biotechnology, Dallas, TX, USA) (1:100) or rabbit anti-Notch2 cleaved N-terminus IgG (Merck Millipore, Darmstadt, Germany) (1:100) overnight at 4 °C in a humidified chamber. After washes, sections were detected with biotinylated rabbit anti-goat IgG (1:500) (Dako, North Sydney, Australia) to detect anti-TNFα antibody and swine anti-rabbit IgG (1:500) (Dako, North Sydney, Australia) to detect anti-Notch2 IgG for 60 min, followed by incubation with streptavidin-HRP (1:700) (R&D systems, Minneapolis, MO, USA) for 60 min and finally detection with DAB Plus chromogen (Dako, North Sydney, Australia). All sections were then counterstained with haematoxylin and analysed as described before [40]. 

### 2.5. Micro-CT Quantification of Vasculature and Lacunar Porosities in Metaphyseal Cortical Bone

In order to visualise cortical bone vasculature canal and lacunae networks, also as a means of quantifying cortical vascular and lacunar canal porosities, tibia bones were scanned using micro-CT scanner (SkyScan 1276, Bruker, Kontich, Belgium) with an X-ray source working at 55 kV/72 µA. Tibial samples were kept in 80% ethanol, fixed on the scanning bed with same settings for all samples (pixel size of 6.5 µm, 0.4° rotation step and 0.25 mm aluminium filter). Cross sectional images were generated from X-ray projections using reconstruction software NRecon (Skyscan, Bruker, Belgium). Vasculature canals were considered as open pores and as vasculature circulates in the cortical bone. In contrast, osteocyte lacunae were considered as close pores as they are completely embedded within bone matrix. Vascular and lacunar canals in the cortical bone were assessed by CTAn software (v.1.15.4.0, Skyscan, Bruker, Belgium) after selecting the region for analysis in metaphyseal region of tibia starting 1 mm below the growth plate and with a height of 2 mm (consisting mainly of secondary spongiosa). The region of interest from the cortical bone was segmented in custom processing by selecting global thresholding and cortical open pores were identified and computed by “sphere fitting” algorithm as described [42]. Cortical vascular canal porosity (Ca.V/TV, %), vascular canal number (Ca.No/mm), canal separation (Ca.Sp, mm) for vasculature analyses and osteocyte lacunar porosity (Lc.V/TV, %) and lacunar number (Lc. No/mm) were quantified using CTAn software and 3D images were constructed using CTvol (Skyscan, Bruker, Belgium). 

### 2.6. Cell Culture Study

Primary rat bone marrow-derived endothelial cells (BMECs) (Cell biologics, Chicago, IL, USA) were grown in endothelial cell complete medium M1266 (Cell biologics) in flasks pre-coated with gelatine-based coating solution (Cell Biologics). To determine the changes in Notch signalling pathway following MTX treatment, BMECs were treated with MTX (10 µM) or saline for 24 h and then were harvested for RNA isolation and gene expression analyses. To investigate the possible role of anti-Notch2 (NRR2) antibody in protecting endothelial cells against MTX damage, cells (at reaching 70% confluency) were treated with saline or MTX (10 µM) along with either the anti-ragweed antibody as control IgG (10 µg mL^−1^) or the anti-Notch2 antibody (10 µg mL^−1^) for 24 h, after which time the cells were harvested and conditioned medium was collected. The selected dose for MTX treatment in vitro is the chemotherapy relevant dose for MTX in cell culture studies [17,43,44]. The dose chosen for anti-Notch2 antibody treatment has previously been used in different in vitro studies to block Notch2 receptor activity effectively [36,44,45]. 

### 2.7. Matrigel Tube Formation Assay

To examine treatment effects on tube formation ability of rat BMECs, a Matrigel tube formation assay was performed. Briefly, in a 96-well plate coated with 80 µL of the Falcon Matrigel Basement Membrane Matrix (In Vitro Technologies, Melbourne, VIC, Australia), BMECs from each group (flask) were serum starved for 1.5 h and then were seeded on Matrigel at 2.5 × 10^4^ per well in M1266 serum free medium. Cells were incubated at 37 °C and 5% CO_2_ for 5 h. The Olympus CKX41 microscope and DP2-SAL Olympus software (Olympus, Melbourne, VIC, Australia) were used for capturing culture images. Tube formation was analysed with Image J software for quantifying numbers of branches [40]. 

### 2.8. Trans-Well Migration Assay

To assess treatment effects on migration ability of rat BMECs, 24-well plate transwell inserts (Costar Permeable Supports, Corning, NY, USA) with 8.0 polycarbonate membrane were used as described [46]. Before cell seeding, inserts were treated with gelatine-based coating solution, then endothelial cells that had been pre-serum-starved for 1.5 h were seeded at a density of 2.5 × 10^4^ cells/insert with 250 µL M1266 serum-free medium. For cells to be able to migrate to the bottom side of inserts, 750 µL M1266 complete medium was added to each lower chamber. Plates were incubated for 16 h at 37 °C and 5% CO_2_. Then, medium was gently removed from each insert and cells were fixed with 70% ethanol for 10 min. After removing ethanol, cells were air-dried for 15 min prior to staining with 0.2% crystal violet for 10 min and then gentle washing for 3 times with PBS. Non-migrated cells were scrapped off with cotton swabs and images were captured by Olympus CKX41 microscope with DP2-SAL Olympus software (Olympus, Melbourne, VIC, Australia). Migrated cells were counted using Image J software. 

### 2.9. Measurement of Nitric Oxide

To investigate bioavailability of nitric oxide (NO), nitrite/nitrate concentrations in BMEC-conditioned culture media following 24 h of MTX±Anti-Notch2 antibody treatment were measured by the Griess assay [47]. Using a Griess reagent kit (Life Technologies, Mulgrave, VIC, Australia), equal volumes of sulfanilic acid and N-(1-naphthyl)ethylenediamine were mixed together to form the Griess reagent and then 20 µL of this Griess reagent was added to each well of 96-well plate containing 150 µL of conditioned medium plus 130 µL of Mili-Q water. The plate was incubated for 30 min at room temperature in the dark prior to absorbance reading at 570 nm. 

### 2.10. Measurement of Pro-Inflammatory Cytokine TNFα in Rat Serum

Serum was collected following cardiac puncture after sacrificing rats, and stored at −80 °C, and enzyme-linked immunosorbent assay (ELISA) was conducted to quantify TNFα in serum of control and treated rats using rat TNFα ELISA kit (Invitrogen, VIC, Australia). 

### 2.11. RNA Isolation and Gene Expression Analyses

Total RNA was isolated from frozen metaphyseal bone samples (containing bone and bone marrow) and cultured BMECs as described before using total RNA isolation kit GeneElute (Sigma-Aldrich, Castle Hill, NSW, Australia) [39,48]. Using iScript Select cDNA synthesis kit (Bio-Rad, Hercules, CA, USA), cDNA was synthesised and quantitative real time PCR assays were conducted with CFX connect PCR machine (Bio-Rad, Hercules, CA, USA) using Sso Advanced Universal SYBR Green Supermix kit (Bio-Rad, Hercules, CA) with primers (Table 1) designed with PRIMER-BLAST (NCBI, Bethesda, MD, USA) and supplied by Sigma-Aldrich (Castle Hill, NSW, Australia) for Notch pathway genes and by Gene-Works (Adelaide, SA, Australia) for other genes. Relative expression was calculated using comparative Ct (2^−ΔCt^) method against Cyclophilin A gene (*PPIA*) as housekeeping gene [49].

### 2.12. Statistical Analyses

Data are expressed as mean ± SEM and analysed with student’s *t* test or standard one-way ANOVA with a Tukey’s multiple comparison test using GraphPad Prism (8.3.0 for Windows, GraphPad Software, San Diego, CA, USA). Significance was considered when *p* < 0.05. 

## 3. Results

### 3.1. MTX Damaging Effect on Bone Marrow Vasculature Is Associated with Upregulation of NICD2, Notch Ligand Jag1 and Notch Target Gene Hey1 in Bone

**Figure 1:**
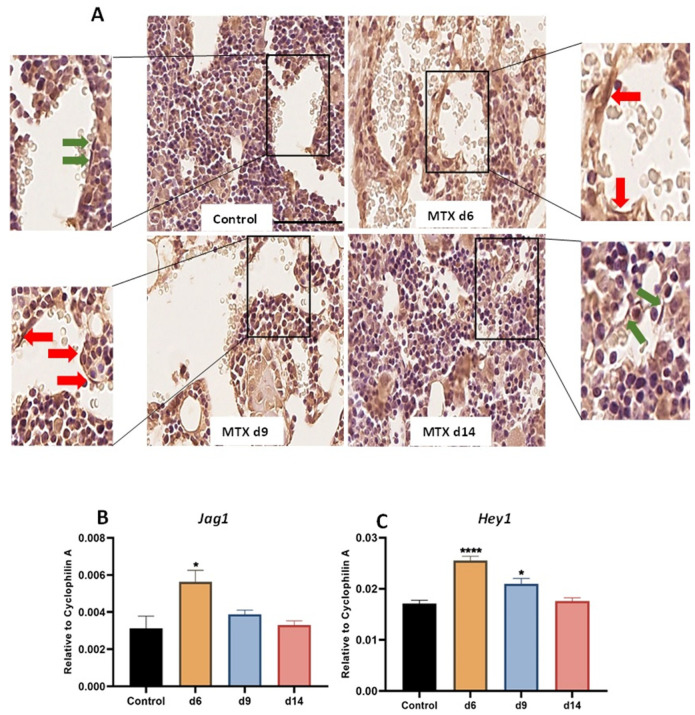
MTX chemotherapy-altered Notch2 intracellular domain (NICD2) protein level, Notch ligand *Jag1* and Notch target gene *Hey1* mRNA expression levels in tibial metaphysis bones. (**A**) Immunohistochemistry staining against NICD2 in tibial secondary spongiosa revealed more abundant NICD2-positive bone marrow endothelial cells (BMECs) (pointed by red arrows) at days (d) 6 and 9 (after the first MTX injection) compared to the control, and more NICD2-negative endothelial cells (pointed by green arrows) at day 14. Scale bar is 50 µm. Quantification real time PCR analyses (using RNA isolated from metaphyseal bone specimens) for (**B**) Notch ligand *Jag1* and (**C**) Notch target gene *Hey1*. * *p* < 0.05 and **** *p* < 0.0001 compared with the control group.

Previous cellular and pathological findings revealed damaging effects on bone and bone marrow vasculature following MTX chemotherapy in rats, particularly at day nine after the first MTX injection (a time point known to have the most significant histological damage) [17,33]. Very recently, we found that elevated expression of Notch2 mRNA (by RT-PCR) and Notch2 protein (by Western blotting) in bone is associated with bone damage in rats, and that supplementary treatment with Notch2-specific antibody against the negative regulatory region of Notch2 receptor (NRR2) protected trabecular bone from MTX-induced damage [50]. In the current study, as a first step to determine whether MTX treatment alters NICD2 protein expression in endothelial cells in bone, immunohistochemical staining was conducted using tibia sections collected from different time points after MTX treatment. As illustrated in Figure 1A (red arrows indicating NICD2-positive and green arrows showing negative endothelial cells), despite some other cells (e.g., bone marrow cells) showing NICD2-positivity, there were obviously more NICD2-positive endothelial cells at days 6 and 9 when compared to control, which appeared to have returned to control level at day 14 (a time point which was previously shown with vasculature recovery following MTX treatment in rats) [17]. 

Gene expression analyses of the Notch ligand (Jag1) and Notch target gene (Hey1) in tibial metaphyseal bone revealed significant upregulation in the mRNA level of *Jag1* (*p* < 0.05 versus the control group) at day 6, which declined to control levels at days 9 and 14 (Figure 1B). Consistent with Notch2 upregulation observed above, there was a remarkable upregulation in the Notch target gene (*Hey1*) at day 6 (*p* < 0.0001) and day 9 (*p* < 0.05) when compared to control, which returned to the control level at day 14 (Figure 1C).

### 3.2. Anti-Notch2 Antibody Treatment Attenuated MTX-Induced Bone Marrow Sinusoidal Dilation 

**Figure 2:**
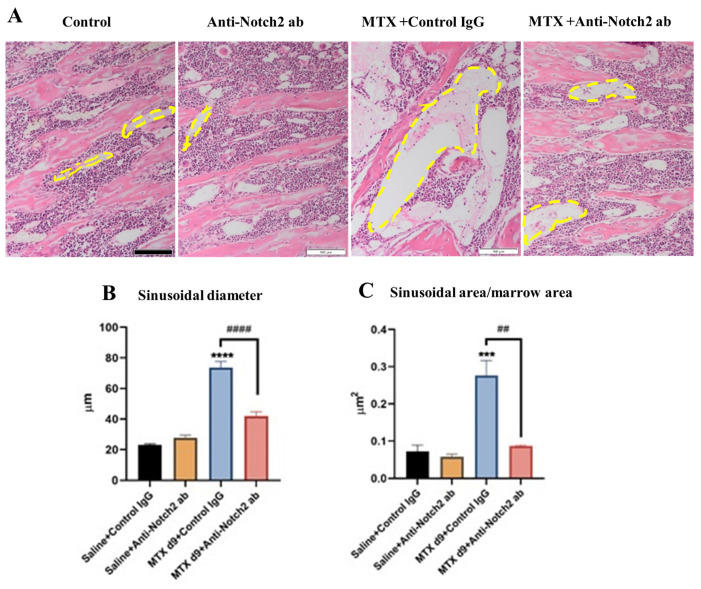
Effect of MTX or MTX+Anti-Notch2 antibody (ab) treatment on bone marrow sinusoid dilation in rats (histological analyses). (**A**) Representative tibial sections with H&E staining from different treatment groups, showing treatment effects on bone marrow sinusoids (indicated by yellow dash lines). Scale bar is 100 µm. (**B**) BM sinusoidal diameters at different treatment groups. (**C**) Treatment effects on sinusoidal area per bone marrow area. *** *p* < 0.001 and **** *p* < 0.0001 compared to control group; ## *p* < 0.01 and #### *p* < 0.0001 compared with MTX+Control IgG-treated group. All the measurements are expressed as mean ± SEM.

As a means of determining treatment effects on bone marrow sinusoidal vasculature damage and recovery, we analysed tibial histological images from the control and treatment groups. Irregular shaped mono-layer thin-walled micro-vessels in the lower secondary spongiosa of metaphysis bone have been considered as sinusoids [16,17]. Tibial histological image analyses illustrated a very significant (*p* < 0.0001) sinusoidal dilation in the lower secondary spongiosa at day 9 following the first MTX treatment in MTX+Control IgG group compared to the control (Figure 2A,B). However, anti-Notch2 treatment remarkably attenuated MTX-induced sinusoidal dilation, when compared to MTX+Control IgG group (*p* < 0.0001) (Figure 2B). Furthermore, the same trends of treatment-induced changes were observed in sinusoidal area per marrow area. There was remarkable increase in the size (area) of vasculature per marrow area in MTX+Control IgG group (*p* < 0.001) compared to control, which was prevented by the anti-Notch2 antibody supplementation (*p* < 0.01) when compared to MTX+Control IgG group (Figure 2C). 

### 3.3. Micro-CT Assessments of Treatment Effects on Vasculature and Osteocyte Lacuna in Cortical Bone 

**Figure 3:**
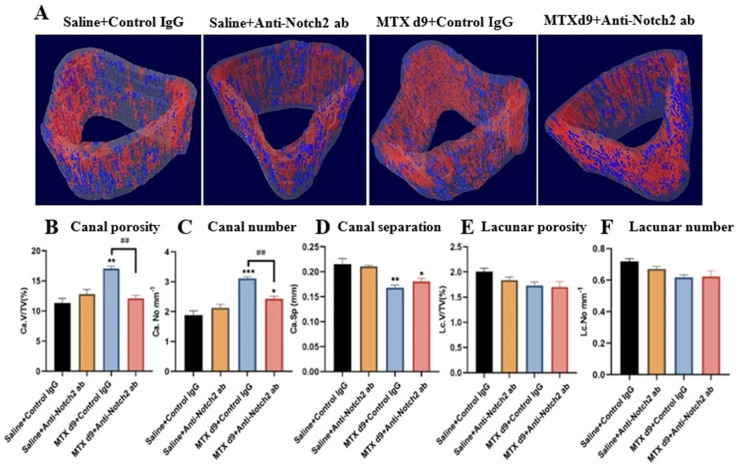
Treatment effects on tibial metaphyseal cortical bone vasculature canals and osteocyte lacuna porosities at day nine after initial MTX dose. (**A**) Representative micro-CT 3D images form control and different treatment groups illustrating the segmentation of the cortical bone vasculature canals (red) and osteocyte lacuna network (blue). (**B**) Vascular canal porosity (Ca.V/TV, %). (**C**) Average vascular canal number (Ca.No, per mm). (**D**) Vascular canal separation (Ca.Sp, mm). (**E**) Osteocyte lacunar porosity (Lc.V/TV, %). (**F**) Osteocyte lacunar number (Lc.No, per mm). All measurements expressed as mean ± SEM. * *p* < 0.05, ** *p* < 0.01 and *** *p* < 0.001 compared to the control; and ## *p* < 0.01 compared between MTX treatment groups.

Micro-CT 3D morphological assessment of vascular canals is considered a well-accepted method of evaluating vasculature density in the cortical bone [51,52]. To determine MTX +/− anti-Notch2 antibody treatment effects on vasculature canals in the tibial metaphysis cortical bone, micro-architecture measurements were performed using micro-CT scans of the bone specimens (Figure 3A). There was a significant increase in the volume of cortical vasculature pores in the MTX+Control IgG treatment group compared to the control group (*p* < 0.01). However, blockade of Notch2 signalling with the anti-Notch2 antibody along with MTX treatment notably attenuated MTX-induced increase in the vascular canal porosity (*p* < 0.01) (Figure 3B). Moreover, a remarkable increase in the number of vasculature canals was found in the MTX+Control IgG treatment group (*p* < 0.001 versus the control group), which was significantly attenuated by the MTX+Anti-Notch2 antibody combination treatment (*p* < 0.01 versus MTX+Control IgG group) (Figure 3C). Moreover, while MTX+Control IgG treatment significantly reduced vasculature canal separation (*p* < 0.01 versus control), this change was found to be less notable in MTX+Anti-Notch2 antibody group (*p* < 0.05 versus the control group) (Figure 3D). Consistent with micro-CT observations, assessments of vasculatures in H&E-stained tibial cortical bone sections also showed similar trends of changes (data not shown). 

In order to investigate whether the changes in the cortical bone vasculature are specific or as a consequence of general alterations in bone matrix porosity, we also quantified close pores (considered as osteocyte lacunae) in the cortical bone [12]. There were no significant alterations in lacunar porosity and lacunar density between the control and treatment groups (Figure 3E,F).

### 3.4. Notch2 Blockade Alters mRNA Expression of Notch Target Gene Hey1 and Attenuates MTX-Induced Increases in Inflammatory Cytokine TNFα Levels

**Figure 4:**
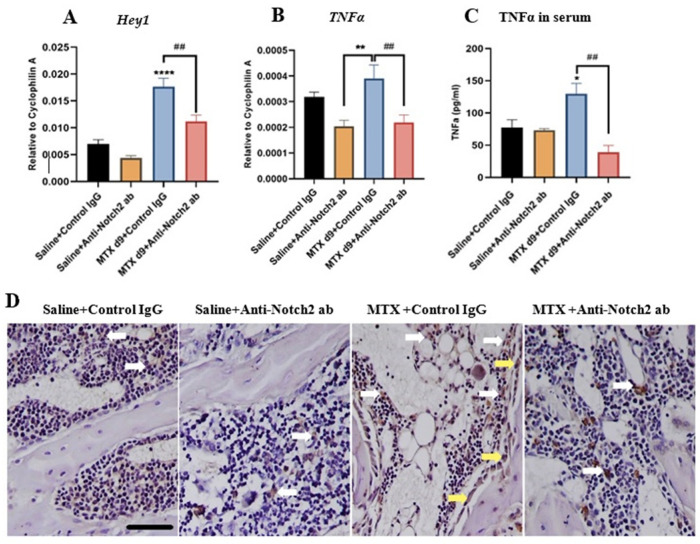
Effects of anti-Notch2 antibody (ab) treatment on expression of Notch target gene Hey1 and inflammatory cytokine TNFα in bone and serums of rats on day (d) nine after the first MTX injection. (**A**) *Hey1* mRNA expression levels as evaluated by RT-PCR. (**B**) *TNFα* mRNA expression levels in bones. (**C**) TNFα protein levels in serum (pg/mL) as assessed by ELISA. (**D**) Representative images (taken from the lower region of secondary spongiosa of metaphysis bone) for TNFα immunostaining of rat bone and bone marrow from various treatment groups, with MTX+Control IgG group illustrating more abundant positivity (bone marrow cells (white arrow heads) and osteoblasts (yellow arrow heads) when compared to the MTX+Anti-Notch2 antibody treatment group. Scale bar is 50 µm. * *p* < 0.05, ** *p* < 0.01 and **** *p* < 0.0001 compared to the control or the anti-Notch2 antibody alone treatment control, and ## *p* < 0.01 when compared between MTX treatment groups.

Our recent data revealed the significant deregulation of Notch2 expression in bone following MTX treatment [50] as well as in endothelial cells as shown above, and the Notch target gene Hey1 has been previously shown to be upregulated with Notch2 activation in endothelial cells following inflammation [27] or following ionizing irradiation [53], and to play a crucial role in vasculature development [54]. Therefore, we have investigated the Notch2 blockade treatment effects on Hey1 gene expression level using RNA isolated from metaphyseal bone specimens. RT-PCR analyses revealed the same change trends in as Notch2 expression, with significant upregulation of Hey1 in MTX+Control IgG group compared to the control (*p* < 0.0001), which was notably attenuated with anti-Notch2 IgG supplementation when compared to MTX+Control IgG group (*p* < 0.01) (Figure 4A). 

Since bodies of evidence have illustrated a direct correlation between TNFα and the Notch2 signalling pathway that controls endothelial cell functionality and survival [21,27], and Notch signalling plays an active role in controlling inflammatory responses and cytokine production [55,56], here we have examined treatment effects on TNFα expression. Quantitative RT-PCR results revealed a significant increase in TNFα mRNA expression in bones at day nine following MTX treatment when compared to the anti-Notch2 treatment alone control group (*p* < 0.01). Interestingly, Notch2 neutralising antibody treatment remarkably reduced TNFα mRNA expression when compared to MTX+Control IgG group (*p* < 0.01) (Figure 4B). Moreover, ELISA assessments of TNFα protein level in serum of rats illustrated a notable increase in MTX+Control IgG-treated rats at day nine compared to the control group (*p* < 0.05) (Figure 4C), which was significantly suppressed with anti-Notch2 antibody supplementation (*p* < 0.01 versus MTX+Control IgG group) (Figure 4C). Consistently, TNFα immunostaining study showed that bone sections from the MTX+Control IgG group had a higher TNFα positivity in bone marrow cells and osteoblasts compared with both control groups, and that Notch2 blockade in MTX-treated rats reduced TNFα positivity when compared with the MTX+Control IgG group (Figure 4D). 

### 3.5. MTX Treatment Alters Notch Signalling in Cultured Rat BMECs and Blocking Notch2 Attenuates MTX-Induced Notch Target Gene Hey1 Overexpression

**Figure 5:**
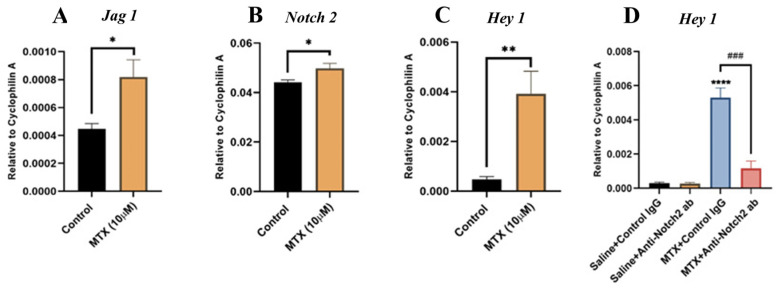
MTX treatment effects on the Notch2 ligand *Jag1*, *Notch2*, and the Notch target gene *Hey1* mRNA expression and the role of Notch2 signalling in MTX-induced *Hey1* induction in cultured rat bone marrow-derived endothelial cells (BMECs). Quantitative real-time PCR gene expression analyses of (**A**) *Jag1*, (**B**) *Notch2*, (**C**) *Hey1*, using RNA isolated from control and MTX-treated (10 µM for 24 h) BMECs. (**D**) Quantitative real time PCR for *Hey1* in BMECs treated for 24 h with Saline+Control IgG, Saline+Anti-Notch2 antibody (ab), MTX+Control IgG, or with MTX+Anti-Notch2 antibody. * *p* < 0.05, ** *p* < 0.01, and **** *p* < 0.0001 compared to control IgG; and ### *p* < 0.001 compared between MTX treated groups. All results are shown as mean ± SEM from three independent experiments.

Next, using rat bone marrow endothelial cell (BMEC) culture models, we aimed to confirm our in vivo observations on the roles of Notch2 signalling in MTX-induced bone vasculature damages and to study its action mechanisms. To examine whether MTX treatment alters Notch pathway in endothelial cells in vitro, cultured rat BMECs were treated with saline or MTX (10 µM) for 24 h prior to RT-PCR gene expression analyses of Notch ligand *Jag1* (known as a more specific ligand for Notch2 receptor) [20] and Notch target gene *Hey1* (previously shown to be associated with changes in Notch2 after vasculature injuries) [21,27]. The results illustrated a significant upregulation in both *Jag 1* (Figure 5A) and *Notch2* (Figure 5B) mRNA expression in MTX-treated BMECs compared to control BMECs (both at *p* < 0.05). Moreover, MTX treatment remarkably upregulated mRNA expression level of the Notch target gene *Hey1* (*p* < 0.01) compared to the control (Figure 5C). 

To evaluate whether MTX-induced *Hey1* mRNA upregulation is associated with Notch2 upregulation, and to investigate whether Notch2 antagonism suppresses the induction of *Hey1* expression, BMECs were treated for 24 h with saline or MTX as above and together with control IgG 10 µg mL^−1^ or with anti-Notch2 antibody 10 µg mL^−1^. Gene expression analyses of *Hey1* again confirmed significant mRNA upregulation for *Hey1* in MTX-treated endothelial cells (*p* < 0.0001) compared to control cells, which was remarkably attenuated with Notch2 blockade (*p* < 0.001 versus MTX+Control IgG group) (Figure 5D). There were no statistically significant differences in *Hey1* expression levels between the anti-Notch2 alone control group and the control IgG alone group. These results suggest that Notch2 blockade inhibits MTX-induced *Hey1* upregulation in rat BMECs. 

### 3.6. Treatment Effects on Migration Ability of Rat BMECs

**Figure 6:**
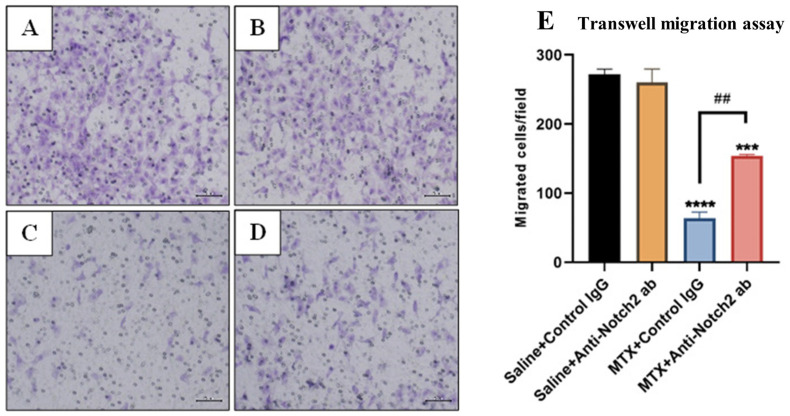
Treatment effects of MTX with/without anti-Notch2 antibody (ab) on migration ability of rat bone marrow-derived endothelial cells (BMECs) as assessed by a transwell migration assay. Representative microscopic images illustrate migrated cells stained with Crystal Violet in (**A**) Saline+Control IgG group, (**B**) Saline+Anti-Notch2 antibody group, (**C**) MTX+Control IgG group and (**D**) MTX+Anti-Notch2 antibody group. Scale bar is 200 µm. (**E**) Average numbers of migrated cells per field (as mean ± SEM) from three independent experiments. *** *p* < 0.001 and **** *p* < 0.0001 compared to control IgG, and ## *p* < 0.01 compared between MTX treatment groups.

To determine the treatment effects on the migration ability of rat BMECs, the transwell migration assay was performed and migrated cells were stained, counted, and expressed as the number of migrated cells per field (Figure 6A–D). Our results revealed that after the BMECs were treated for 24 h with MTX 10 µM+Control IgG (10 µg mL^−1^), their migration ability was significantly reduced (*p* < 0.0001) when compared to control IgG alone group (Figure 6E). While Notch2 blockade did not affect BMEC migration when compared with control IgG alone group, it notably ameliorated the migration ability of MTX-treated cells (*p* < 0.001) when compared with MTX+Control IgG group (Figure 6E). 

### 3.7. Treatment Effects on Endothelial Cell Tube Formation Ability 

**Figure 7:**
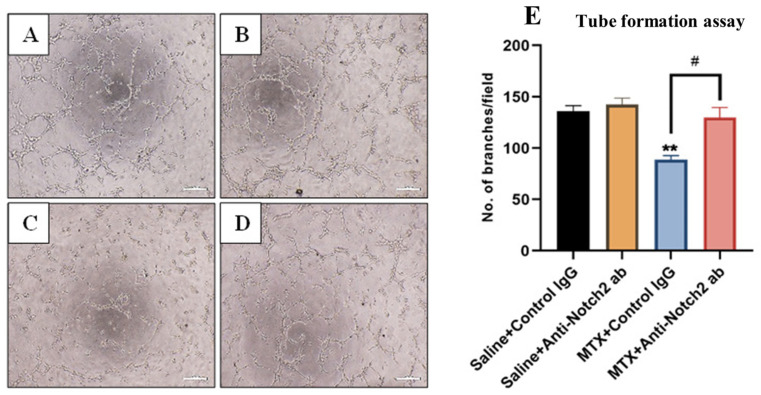
Treatment effects of MTX ± anti-Notch2 antibody (ab) on tube formation ability of rat bone marrow-derived endothelial cells (BMECs). Representative microscopic images of tube formation for endothelial cells treated with (**A**) Saline+Control IgG, (**B**) Saline+Anti-Notch2 antibody, (**C**) MTX+Control IgG, and (**D**) MTX+Anti-Notch2 antibody. Scale bar is 200 µm. (**E**) Average numbers of branches per field (as mean ± SEM) from three independent experiments. ** *p* < 0.01 compared to the control IgG group and # *p* < 0.05 when compared between MTX treatment groups.

Treatment effects on functionality of endothelial cells to make tube-like structures were assessed with Matrigel tube formation assays with results expressed as numbers of branches per microscopic field (Figure 7A–D). When BMECs were treated with MTX+Control IgG for 24 h, tube formation ability was decreased (*p* < 0.01) when compared to control IgG alone treated cells (Figure 7E), which was significantly attenuated with Notch2 blockade (*p* < 0.05 versus MTX+Control IgG group) (Figure 7E). However, there were no statistically significant differences in numbers of branches formed between control IgG and control anti-Notch2 alone-treated cells (Figure 7E). 

### 3.8. Notch2 Blockade Protecting Endothelial Cell Functionality against MTX Damage Is Accompanied by Induction of NO and VEGF 

**Figure 8:**
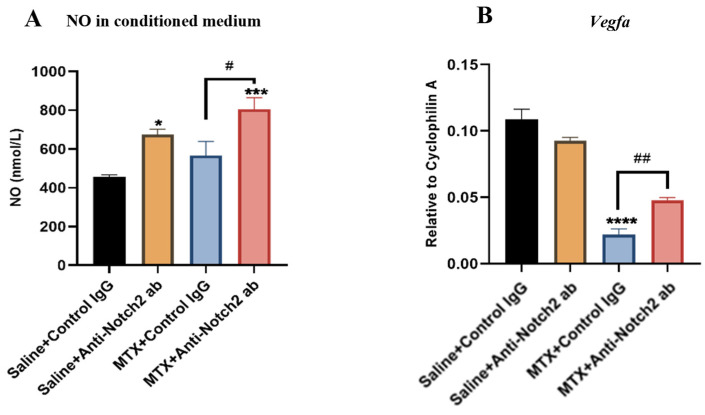
Treatments effect on levels of nitric oxide (NO) in conditioned medium and *Vegfa* mRNA expression in bone marrow-derived endothelial cells. (**A**) Levels of NO in conditioned medium from different treatment groups as evaluated by Griess assays. (**B**) Quantitative real time PCR for *Vegfa* mRNA expression. * *p* < 0.05, *** *p* < 0.001 and **** *p* < 0.0001 compared to the Saline+Control IgG group, and # *p* < 0.05 and ## *p* < 0.01 when compared between MTX treatment groups. Results shown are mean ± SEM from three independent experiments.

The Jag1/Notch pathway was shown to mediate endothelial cell dysfunction via suppressing endothelial nitric oxide (NO) synthase (eNOS) [57]. Nitric oxide is known as a pro-angiogenic factor [58] and a main regulatory molecule for normal and pathological vasculature remodelling [59]. To investigate treatments effect on level of nitric oxide (NO) production, NO levels in BMEC cultured medium were measured. Results indicated a significantly higher level in cells treated with MTX+Anti-Notch2 antibody than cells treated with MTX+Control IgG (*p* < 0.001). Moreover, when control groups were compared, there was a notable increase (*p* < 0.05) following treatment with anti-Notch2 antibody when compared to control IgG treatment (Figure 8A). 

It is well known that VEGF has a crucial role in angiogenesis and vascular homeostasis [60]. VEGF modulates proliferation and migration of endothelial cells [61]. Since cross talk between Notch and VEGF pathways has also been reported, and inhibition of Notch1,4 receptors indirectly modulates VEGF expression in human endothelial cells [62], RT-PCR analyses were performed with RNA isolated from BMECs in different treatment groups to investigate the treatment effects of MTX +/− anti-Notch2 antibody on *Vegfa* mRNA expression level in endothelial cells. Results showed a significant down regulation in *Vegfa* mRNA expression in MTX+Control IgG group compared to the control (*p* < 0.0001), which was significantly attenuated in cells treated with MTX+Anti-Notch2 antibody (*p* < 0.01 versus MTX+Control IgG group) (Figure 8B). No statistically significant differences were observed in *Vegfa* mRNA expression between Saline+Control IgG and Saline+Anti-Notch2 antibody groups (Figure 8B). 

## 4. Discussion

MTX chemotherapy still is a mainstay in cancer treatment regimens for childhood and adult malignancies. However, like many cancer therapeutics, it has detrimental bone marrow and bone long-term side effects in cancer patients and survivors [1,33,39]. Recent findings have shown that MTX chemotherapy increased bone marrow sinusoidal dilation and damage in rats [17]. However, the underlying molecular mechanism for bone marrow micro-vasculature damage and recovery potential following MTX chemotherapy is not clear and there is no protective treatment. Very recently, we have found that deregulation of Notch2 signalling in osteoblasts plays a central role in MTX chemotherapy-induced bone damage [50], and Notch2 has been previously shown to play an important role in inflammation-mediated endothelial dysfunction [21,27]. However, the potential regulatory roles of Notch2 signalling in bone micro-vasculature injury and recovery following MTX chemotherapy remain unknow. Using a rat model, the current study has revealed that increased micro-vasculature dilation and dysfunction following MTX chemotherapy was accompanied by over activation of Notch2 in bone marrow endothelial cells and elevation of production and release of TNFα from osteoblasts and bone marrow cells, that is known to cause endothelial cell dysfunction. Pharmacological blockade of Notch2 using a neutralising antibody was found to ameliorate MTX-induced bone micro-vasculature damage in the bone marrow and cortical bone, which could be directly via suppressing Notch2 signalling in endothelial cells and indirectly by inhibiting Notch2 signalling in osteoblasts and bone marrow cells and thus reducing TNFα production. Our in vitro results also indicated that MTX treatment induced Notch2/Hey1 pathway activation in rat BMEC culture that negatively affects their functionality, and that blockade of Notch2 induced NO production and *Vegfa* mRNA expression and partially restored impaired BMEC functionality caused by MTX.

### 4.1. Notch Signalling Alteration Is Associated with MTX-Induced Bone Micro-Vasculature Damage and Notch2 Blockade Ameliorates MTX-Induced Vasculature Damage in the Bone Marrow and Cortical Bone 

In this study, we used a rat model of MTX chemotherapy which mimics clinical treatment of the major childhood cancer, acute lymphoblastic leukemia. Previously, using the same model of MTX chemotherapy, bone marrow sinusoid dilation and haemorrhage were observed particularly at days 6 and 9 following the first of five once-daily MTX administration with recovery being seen at day 14 [17]. However, the molecular mechanisms for this are not clear. Recently we have found that MTX chemotherapy-induced bone damage was linked with Notch2 upregulation and over-activity in osteoblasts in rats. Notch signalling has a crucial role in vasculature development and homeostasis by controlling differentiation and cell fate decision [63,64], and Notch2 deregulation has been implicated in bone and vasculature abnormalities [18,20]. Previous investigations demonstrated that the induction of Notch2 in endothelial cells following TNFα treatment stimulated pro-apoptotic effects and reduced cell survival, and therefore promoted inflammatory cytokine-mediated vasculature damage [21,27], and the reduced endothelial cell survival was reversed by Notch2 silencing [21]. 

Consistently, the current study found that histological changes in bone marrow vasculatures observed at days 6 and 9 and 14 following MTX chemotherapy were associated with Notch2 signalling deregulation in endothelial cells. Consistent with Notch2 upregulation, Notch ligand Jag1 and Notch target gene Hey1 mRNA expression levels were also found to be elevated, which suggest the implication of increased activation of Notch2 signalling in endothelial cell dysfunction following MTX treatment. Consistent with our findings, Jag1 and Hey1 upregulation following irradiation was shown to negatively affect the functionality of human micro-vascular endothelial cells [53]. In this current study, we have illustrated that Notch2 blockade restored MTX-induced bone marrow vasculature dilation in the bone marrow. Furthermore, investigation into vasculature canals in the cortical bone with micro-CT analysis (which has been suggested as a valid approach for quantification of vascular density in cortical bone [42,51]) revealed that Notch2 antagonism restored MTX-induced increases in vasculature canal volume, porosity and vascular canal number in the cortical bone. Previously, it has been shown that increased vasculature canal volume and porosity in cortical bone reduces blood perfusion and interstitial fluid flow and diminishes bone strength [12,65], and that impaired blood flow in bone is associated with dysregulated Notch signalling in endothelial cells which negatively affects endothelial homeostasis, angiogenesis and, thereby, osteogenesis in the skeletal system [66]. Taken together, the findings from the current study suggest that the overactivation of Notch2 mediates MTX chemotherapy-induced micro-vasculature damage and that blocking Notch2 signalling during MTX treatment can protect the micro-vasculature in the bone marrow and cortical bone in rats.

### 4.2. Notch2 Blockade Alleviates MTX-Induced Increased Levels of Inflammatory Cytokine TNFα in Bone and Serum: A Possible Indirect Mechanism for Protecting Micro-Vasculature 

Increased inflammation as a long-term side effect of cancer chemotherapy and irradiation is well documented, and the release of TNFα from mononuclear cells is known to result in endothelial cell damage following irradiation [67,68]. Previously, MTX chemotherapy-induced bone loss in rats was associated with increased levels of pro-inflammatory cytokines including TNFα in bone and in serum as well as increased activation of nuclear factor kappa B (NF-κB) in skeletal cells [69]. Consistently, in the current study, we found TNFα was upregulated in both mRNA and protein levels in bone and in serum at day 9 following MTX treatment in rats. Previously it was suggested that irradiation-induced inflammatory response changes endothelial cell homeostasis and integrity by induction of cellular adhesion molecules [70]. This inflammatory storm can activate B and/or T lymphocyte response against endothelial cell surfaces [71,72]. In addition, inflammatory cytokines such as TNFα are considered as important mediators in endothelial dysfunction in systemic diseases or pathological conditions where inflammation is involved [73,74]. One of the mechanisms for TNFα-induced endothelial dysfunction could be the vascular barrier dysfunction and the increased vascular permeability [75,76]. Leibovich et al. have shown that TNFα stimulates capillary blood vessel formation in the rat cornea and the developing chick chorioallantoic membrane at very low doses [77] and this could also be the effect on tumour vasculature involved in tumorigenesis. 

Action of both NF-κB and Notch pathways has been found to be required for the expression of interferon gamma in lymphocytes, suggesting the cross talk between both pathways for stimulation of inflammatory response [55]. Activation of Notch signalling has also been illustrated following inflammatory diseases such as rheumatoid arthritis (RA) [78,79] and atherosclerosis [80]. Notch signalling can stimulate T cell proliferation and enhance inflammatory cytokine production [56]. Pharmacological inhibition of Notch attenuated RA in an animal model by suppressing production of proinflammatory cytokines [32]. Other studies showed that induction of Notch2 in endothelial cells in response to TNFα reduces their survival and functionality [21,27]. Our results illustrated that induction of Notch2 in bone and bone marrow endothelial cells after MTX chemotherapy is apparently linked with elevation of TNFα in osteoblasts and bone marrow cells (possibly monocytes/macrophages), which may indirectly induce endothelial cell dysfunction and vasculature damage. Consistent with this, Notch2 blockade was found to reduce TNFα mRNA and protein levels and restored MTX-induced micro-vasculature dilation and dysfunction. 

### 4.3. MTX Treatment Alters Notch Signalling in Cultured Rat BMECs and Notch2 Blockade Mitigates MTX-Induced Damaging Effects on Endothelial Cell Functionality 

To examine the potential direct role of Notch2/Hey1 pathway in endothelial cell dysfunction, we conducted in vitro studies assessing treatment effects with MTX ± anti-Notch2 antibody in cultured rat bone marrow-derived endothelial cells (BMECs). Our results demonstrated that MTX exposure at a clinical chemotherapy relevant dose (10µM) [17,43,44] induced Notch2 activation with significantly increased mRNA expression levels of Notch ligand Jag1, Notch2 receptor, and Notch target gene Hey1 in cultured cells, and that supplementation of anti-Notch2 antibody suppressed MTX-induced Hey1 induction. Previously, over-expression of Hey1 was shown to inhibit proliferation, tube formation and migration in human endothelial cells [81]. Similarly, Hey1 upregulation was illustrated in endothelial cells following irradiation, which reduces migration ability in human micro-vascular endothelial cells, and this was reversed by a pan Notch inhibitor (γ-secretase inhibitor) [53]. 

Many studies have demonstrated that Notch signalling induces a quiescent endothelial cell phenotype by influencing proliferation and migration [82,83,84]. A previous investigation showed that MTX negatively affects rat hepatic sinusoidal endothelial cell functionality [17]. Consistently, our results illustrated that MTX treatment decreases tube formation and migration ability of rat BMECs, which were significantly attenuated by Hotch2 inhibition, suggesting that increased Notch2 activation plays an important role in MTX adverse effects on endothelial cell abilities in tube formation and migration. 

The cross talk between Notch, NO and VEGF pathways control endothelial cell homeostasis and functions [23,85,86]. NO is well known as an angiogenic mediator that maintains vascular homeostasis and protects vasculature from injuries [87,88]. It has a critical role in endothelial cell proliferation and migration [89] and has anti-inflammatory properties in vasculature [90]. While Notch blockade in a tumour’s endothelium results in reduced NO levels [91], it has been reported that Notch overactivity negatively regulates NO levels in healthy hepatic sinusoidal endothelial cells, possibly due to the control of Hes1 or Hey1 [92]. Consistently, the current study showed that anti-Notch2 antibody treatment in BMECs suppressed Hey1 mRNA expression and increased the production of NO in cultured endothelial cells compared to MTX alone, suggesting that increased production of NO, as a potent angiogenic factor, is involved in the protective effect of Notch2 blockade on endothelial cell functionality against MTX adverse effects. 

Notch signalling is implicated in VEGF pathway via a feedback loop (18). Activation of the VEGF-A/VEGFR2 pathway in endothelial cells promotes vasculature growth by induction of Notch signalling [93,94]. Notch inhibition was shown to upregulate expression of VEGF-A, which induces endothelial proliferation and sprouting [94], and loss of Hey1/2 in mouse embryos caused an increase in *Vegfa* mRNA expression level [54]. Consistently, we found that, upon Notch2 blockade, Notch target Hey1 mRNA expression level was drastically declined in treated BMECs, which was accompanied with an increased *Vegfa* mRNA expression level. Our in vitro findings illustrate that MTX-induced damage in endothelial cell functionality is associated with overactivation of the Notch2/Hey1 pathway, and that anti-Notch2 antibody co-treatment with MTX partially protects endothelial cell functionality possibly via increased NO production and *Vegfa* expression in BMECs. 

## 5. Conclusions

**Figure 9:**
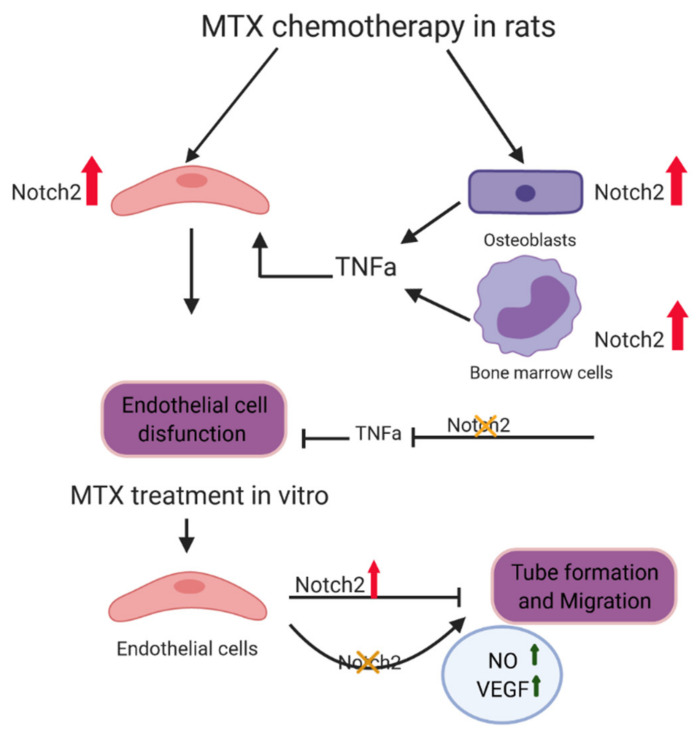
Schematic representation of function of Notch2 activation in endothelial cell and bone vasculature damages following MTX treatment. Activation of Notch2 in osteoblasts and bone marrow cells after MTX treatment may result in increased production and release of tumour necrosis factor alpha (TNFα), which, together with Notch2 overactivation in endothelial cells, may cause vasculature dysfunction and vasodilation. Notch2 antagonism can significantly attenuate MTX treatment-induced micro-vasculature damage. MTX treatment in vitro also induces Notch2 pathway in cultured rat bone marrow endothelial cells (BMECs), which is linked with decreased tube formation and migration ability; and anti-Notch2 antibody treatment ameliorates MTX-induced adverse effects on BMEC functionality, which is possibly due to increased production of nitric oxide (NO) production and expression of vascular endothelial growth factor (VEGF) in endothelial cells.

The results of this study suggest a crucial role of increased Notch2 signalling involving complex mechanisms of action in MTX chemotherapy-induced bone micro-vasculature damage in rats. Following MTX chemotherapy, there was Notch2 overactivation in endothelial cells in bone, and induction of Notch2 activity in osteoblasts and bone marrow cells may induce the expression and release of TNFα, an inflammatory cytokine known to cause endothelial cell damage. Together, these direct and indirect effects of Notch2 may cause endothelial cell damage and vasculature impairment in both bone marrow and in the cortical bone, effects that can be attenuated by Notch2 signalling blockade. Consistently, MTX treatment induced the activation of Notch2/Hey1 pathway in cultured rat bone marrow-derived endothelial cells and reduced their tube formation and migration ability, effects that were partially prevented by Notch2 blockade. These in vitro findings support the direct impact of Notch2 overactivity in endothelial cells. Furthermore, our study suggests that targeting Notch2 attenuates MTX side effects on bone micro-vasculatures indirectly via controlling TNFα storming, and that it also may ameliorate endothelial cell functionality directly by induction of NO and VEGF in these cells (Figure 9). Our findings have thus illustrated an important role of Notch2 signalling in mediating MTX chemotherapy-induced vasculature damage and suggest that Notch2 could be a potential therapeutic target to protect bone micro-vasculature against MTX adverse effects. Since the roles of Notch2 overactivity and the potential of its targeting in haematological malignancies [95] and breast cancer [96] have been previously reported, it might be reasonable to consider Notch2 targeting as a part of treatment regimen against these type of cancers as well as a means of protecting bone micro-vasculature during chemotherapy, a possibility which needs further investigation.

## Figures and Tables

**Table 1:** Primers used in this study.

Gene	Forward Primer (5′-3′)	Reverse Primer (5′-3′)
*Cyclophilin A*	GAGCTGTTTGCAGACAAAGTTC	CCCTGGCACATGAATCCTG
*Hey1*	GGAGAGCGCAGACGAGAATG	CTCGATGATGCCTCTCCGTC
*Notch2*	ATGCCGGGTTTCAAAGGTGT	ATGTCGATCTGGCACACTGG
*Jagged-1*	CATCGGGGGCAATACCTTCA	GCAAAGTGTAGGACCTCGGC
*VEGF-A*	ATCTTCAAGCCGTCCTGTGTG	TGAGGTTTGATCCGCATGATC
*TNFa*	ATGGCCCAGACCCTCACACTCAGA	CTCCGCTTGGTGGTTTGCTACGAC

## Data Availability

The data that support the findings of this study are available on reasonable request from the corresponding author.
